# Unraveling the Molecular Mechanism of Pre-mRNA Splicing From Multi-Scale Simulations

**DOI:** 10.3389/fmolb.2019.00062

**Published:** 2019-08-06

**Authors:** Lorenzo Casalino, Alessandra Magistrato

**Affiliations:** ^1^Department of Chemistry and Biochemistry, University of California, San Diego, La Jolla, CA, United States; ^2^Consiglio Nazionale delle Ricerche–Istituto Officina dei Materiali, International School for Advanced Studies (SISSA), Trieste, Italy

**Keywords:** splicing, spliceosome, group II introns, molecular dynamics, QM/MM

## Introduction

The removal of non-coding introns within a precursor messenger RNA (pre-mRNA) transcript is a key step of gene expression and regulation, occurring via two transesterification reactions mediated by at least two Mg^2+^ ions (Kastner et al., 2019). Whereas in lower organisms this process is self-regulated by group II intron ribozymes (G2IRs) performing their own excision from a pre-mRNA strand, in eukaryotes, due to the increased complexity of the genome, these autocatalytic RNAs have evolved into a majestic protein/RNA machinery—the spliceosome (SPL)—composed of hundreds of proteins and five small-nuclear (sn)RNA filaments (Marcia and Pyle, 2012; Yan et al., 2019). The SPL, acting as a protein-directed metallo-ribozyme, promotes the conversion of pre-mRNAs into mature mRNAs. This massive architecture revolves around its central core constituted by Spp42/Prp8 protein (*S. Pombe/S. Cerevisiae* or human, respectively) and a catalytic site fully resembling that of G2IRs (Yan et al., 2019). As the most eminent genome tailor, the SPL undergoes a relentless compositional and conformational remodeling, repetitively assembling and transforming at every splicing cycle into eight distinct complexes (A, B, B^act^, B^*^, C, C^*^, P, ILS) to achieve splicing with a single nucleotide precision.

Recent developments in single-particle cryo-EM have led to elucidate a plethora of near-atomic resolution structures of SPL complexes from human and yeast strains, thus allowing decades of biochemical, structural and functional studies to be interpreted. In this context, multiscale simulations can contribute to deciphering the intricacies of the splicing mechanism by assessing the chemical details of the pre-mRNA cleavage, and the role of the extraordinarily convoluted protein/RNA environment in creating the appropriate structural scaffold that finely modulates introns removal (Yan et al., 2019). Nevertheless, the size and the inner complexity of the SPL machinery require a wise use of advanced multiscale simulations to tackle the many different peculiarities of its mechanism, as shown in the following showcased studies.

## Chemical Mechanism of Pre-mRNA Splicing in Prokaryotes

The structure of the SPL catalytic site, impressively similar to that of its evolutionary predecessor G2IRs, is well-preserved among the distinct structures that have been solved. A series of crystal structures from *Oceanobacillus iheyensis* captured group IIC intron at sequential stages of the catalytic process, allowing a first structural breakthrough for unraveling the chemical mechanism of pre-mRNA splicing (Marcia and Pyle, 2012). These crystallographic reconstructions revealed an active site containing a four-metal-ion cluster made of two Mg^2+^ and two K^+^ ions, the former being catalytically active, while the latter most likely playing a structural role. Building on these structures, classical and hybrid quantum-classical QM/MM simulations enabled the investigation of the first and rate-determining step of the splicing reaction as promoted by G2IRs (Casalino et al., 2016). In particular, this work focused on the water-mediated 5′-exon cleavage mechanism (hydrolytic path). In fact, in G2IRs the hydrolytic catalysis can be as operative as the branching pathway, where, instead, the reaction is started by a conserved bulged adenosine within the branch point sequence (BPS). By using classical and QM(Car–Parrinello)/MM molecular dynamics (MD), with the QM part described at Density Functional Theory (DFT)-BLYP level of theory, and the MM part treated with the AMBER- ff12SB (ff99+bsc0+χOL3) force field (FF) (Pérez et al., 2007; Zgarbová et al., 2011; Maier et al., 2015), in combination with thermodynamic integration to enable the reaction event within the limited time-scale of the QM/MM MD simulations, this study unveiled a novel dissociative two-Mg^2+^-ion mechanism in which the bulk water acts as general base (Casalino et al., 2016).

The two-Mg^2+^-ion motif is a well-established catalytic cofactor shared by many enzymes processing nucleic acids. In these enzymes, the phosphodiester bond hydrolysis is believed to occur according to the Steitz and Steitz's proposal. In its original postulation, confirmed by distinct computational studies, the two Mg^2+^ ions act as Lewis acids activating the nucleophile, stabilizing the leaving group and the transition state (Palermo et al., 2015; Sgrignani and Magistrato, 2015). At variance with this, in G2IRs a dissociative mechanism takes place, with the reactive water detaching from the Mg^2+^ ion and performing the attack on the scissile phosphate while still in its non-deprotonated form. Only after the nucleophilic substitution has started, the catalytic water eventually releases its proton to the bulk water and terminates the reaction. In this mechanism one Mg^2+^ ion activates the scissile phosphate group by making it more electrophilic, while the second Mg^2+^ stabilizes the leaving group. Hence, in this chemical path the role of the two Mg^2+^ ions remarkably differs from that of protein enzymes performing a two-metal-aided catalysis. It is tantalizing to believe that this mechanism may be specific for ribozymes, where the catalytic site is exclusively formed by the RNA sugar–phosphate backbone bearing a lower specificity/efficiency to promote the reaction than that of enzymes. This peculiar mechanism may represent an ancestral version of the two-Mg^2+^-ion catalysis later evolved in enzymes and in protein-directed ribozymes (spliceosome) (Casalino et al., 2016).

## Splicing Mechanism Modulation by the Protein Environment

**Figure 1:**
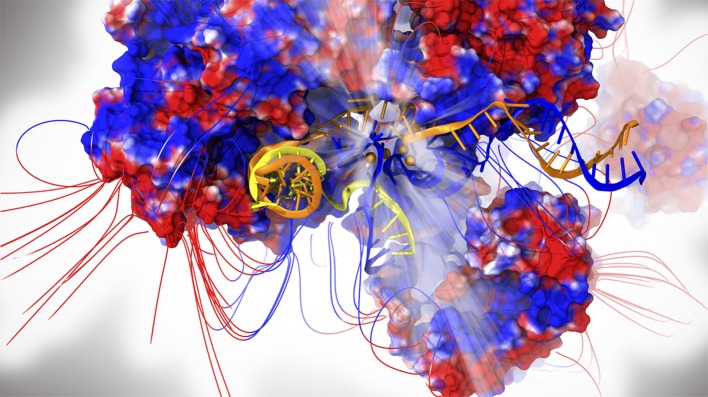
The intron lariat spliceosome complex. Proteins are shown with electrostatic surface (blue/red colors for positive/negative charges, respectively) together with the respective field lines. The intron lariat (yellow), U2 (orange) and U6 snRNA are represented as cartoon. Mg^2+^ ions are depicted as orange spheres. The catalytic center is highlighted by light rays.

In spite of the large number of cryo-EM structures of the SPL published as of yet, no catalytically competent form has been trapped, thus hampering a study of the chemical mechanism of splicing in eukaryotes. Moreover, the large size and complexity of the SPL pose serious challenges even when attempting to unravel its functional properties. Indeed, the deposited cryo-EM maps usually have a resolution ranging between 3 and 4 Å in the core and even reaching lower values in the peripheral regions of the macromolecular assembly, which often displays structural gaps (Kastner et al., 2019; Plaschka et al., 2019; Yan et al., 2019). For these reasons, in order to perform all-atom simulations of the SPL it is mandatory to find a compromise between system size and accuracy. In the first MD simulation study published to date, based on the first near-atomistic SPL structure solved from yeast *S. Pombe* capturing the intron lariat spliceosome (ILS) complex (Yan et al., 2015), two explicitly solvated core model-systems containing ~1,000,000 atoms were built and simulated via multi-replica MD simulations for a cumulative statistics of few microseconds (Figure 1). In these simulations the AMBER-ff12SB FF was used for proteins (Maier et al., 2015), whereas ff99+bsc0+χOL3 FF was adopted for RNAs (Pérez et al., 2007; Zgarbová et al., 2011).

Correlation analyses, principal-component analysis (PCA), and electrostatic calculations disentangled the cooperative motions underlying the SPL functional dynamics, unraveling the role of electrostatics in modulating these movements (Casalino et al., 2018). The simulations provided unprecedented insights on the SPL functional plasticity, assigning to Spp42 (Prp8 in human) a central role in finely directing the motions of many distinct SPL components. Metaphorically, the resulting scenario is that of Spp42 as an orchestra conductor of the gene regulation symphony. The essential dynamics extracted from the PCA revealed, consistently with the stage of the splicing cycle investigated, an electrostatically-driven displacement and unrolling of the U2/intron-lariat branch helix co-promoted by Cwf19 (CWF19L2 in human) and Spp42, both involved in the ILS disassembly (Casalino et al., 2018). Strikingly, the implication of Cwf19 in the branch helix unwinding was thereafter corroborated by recent cryo-EM studies on the human SPL (Zhang et al., 2019). Despite the intrinsic limitations of this study due to the large size of the system and the well-known flaws of RNA (Šponer et al., 2018) and Mg^2+^ (Casalino et al., 2017) FFs, this study has opened new avenues for probing this incredible machinery with atomic-level simulations.

## Discussion

A detailed comprehension of the molecular terms of eukaryotic splicing has entailed implications for revolutionary gene modulation therapies and drug discovery studies aimed at fighting the over 200 human diseases associated with splicing defects. Upon the deposition of the first SPL structure from yeast in 2015, many human cryo-EM maps have been solved, thus opening new opportunities to dissect detailed aspects of this machinery (Kastner et al., 2019; Plaschka et al., 2019; Yan et al., 2019). Among the unmet questions that need to be solved from an atomic-level perspective, the molecular recognition mechanism by which SPL can recruit key intronic sequences at the 3′ and 5′ splice sites, as well as that of the conserved BPS, stands out. The subtle molecular foundations ensuring the reliable identification of authentic consensus splice sites (constitutive splicing), while simultaneously providing some flexibility in the selection of non-consensus ones (alternative splicing) remain unclear. Deregulated constitutive/alternative splicing is well-known to lead to aberrant mRNA transcripts, which may either induce non-sense mediated decay or result in functionally-altered proteins, deleteriously affecting cells functions. In this context, research efforts have been devoted to understanding the mechanism by which mutations of the splicing factor SF3B1 affect BPS recognition, thus leading to aberrant splicing and to the outbreak of distinct hematological malignancies (Cretu et al., 2018). Splicing modulators hitherto trapped in SF3B1 have been found to target the BPS recognition site, elucidating the structural basis of their inhibition mechanism (Cretu et al., 2018; Zhang et al., 2018). Large-scale genomics studies have recently indicated that splicing abnormalities and cancer onset are strongly entwined. Thus, while eagerly awaiting for more structures to be released in the forthcoming years, we expect SPL to become an increasingly important subject of drug design studies tackling distinct types of cancer.

Although the reported results from all-atom simulations—and all the possible future applications—appear to be very encouraging (Casalino et al., 2018; Palermo et al., 2019), several challenges need to be tackled, starting from the amelioration of current RNA and protein/RNA FFs (Šponer et al., 2018). Moreover, even though we have assisted to a fast development of computer hardware and software allowing for brute force unbiased MD simulations, biologically relevant time scales remain computationally extremely demanding and out of reach to most computational labs. In this respect, enhanced sampling and free energy methods to study rare events taking place in complex biological contexts call for further improvements (Miao and McCammon, 2016; Valsson et al., 2016). The presence of metals within the catalytic core of the SPL, which in fact makes it a protein-directed metallo-ribozyme, poses serious difficulties for a reliable fully classical prediction of its properties (Vidossich and Magistrato, 2014; Brunk and Rothlisberger, 2015). For this reason, the use of highly parallel QM/MM MD schemes capable of better exploiting large computational infrastructure would be ideal (Bolnykh et al., 2019; Olsen et al., 2019). A timely fashion communication between the QM and MM would in fact allow more efficient QM(DFT)/MM MD calculations, accounting for larger QM regions and longer simulation time than the accustomed ~100 atoms and ~100s ps time scale, respectively.

In this scenario, we expect that new methodological advances in computer simulations, modeling and analysis techniques will foster atomic-level studies of the SPL, contributing to an utter comprehension of this fundamental step of gene expression. This will also be of service for a better understanding of the allosteric signaling between distal sites, which occurs via the entangled protein/RNA networks characterizing the SPL, and for the discovery of druggable allosteric sites (Palermo et al., 2017). On a final note, we hope that any related breakthrough might help to elucidate the role of splicing pathways in cancer, concretely opening appealing opportunities for creating therapeutic approaches and innovative gene manipulations tools.

## Author Contributions

LC and AM designed research and wrote the paper.

### Conflict of Interest Statement

The authors declare that the research was conducted in the absence of any commercial or financial relationships that could be construed as a potential conflict of interest.
